# Evaluation of Antitumoral Activity in a 3D Cell Model of a Src Inhibitor Prodrug for Glioblastoma Treatment

**DOI:** 10.3390/pharmaceutics17060704

**Published:** 2025-05-27

**Authors:** Letizia Clementi, Federica Poggialini, Francesca Musumeci, Julia Taglienti, Emanuele Cornacchia, Chiara Vagaggini, Anna Carbone, Giancarlo Grossi, Elena Dreassi, Adriano Angelucci, Silvia Schenone

**Affiliations:** 1 Department of Biotechnological and Applied Clinical Sciences, University of L’Aquila, Via Vetoio, 67100 L’Aquila, Italy; letizia.clementi@univaq.it (L.C.); julia.taglienti@student.univaq.it (J.T.); emanuele.cornacchia@graduate.univaq.it (E.C.); 2 Department of Biotechnology, Chemistry and Pharmacy, University of Siena, Via Aldo Moro, 2, 53100 Siena, Italy; federic.poggialini@unisi.it (F.P.); chiara.vagaggini@student.unisi.it (C.V.); elena.dreassi@unisi.it (E.D.); 3 Department of Pharmacy, University of Genoa, Viale Benedetto XV, 3, 16132 Genoa, Italy; francesca.musumeci@unige.it (F.M.); anna.carbone1@unige.it (A.C.); giancarlo.grossi@unige.it (G.G.); silvia.schenone@unige.it (S.S.)

**Keywords:** bioprint, targeted therapy, tyrosine kinase, pyrazolo[3,4-*d*]pyrimidines, anticancer therapy

## Abstract

**Background**: Three-dimensional (3D) cell models may bridge the gap between two-dimensional (2D) cell cultures and animal models. Technical advances have led to the development of 3D-bioprinted cell models, characterized by greater reproducibility and the ability to mimic in vivo conditions. Glioblastoma multiforme (GBM) is a highly aggressive brain tumor with poor clinical outcomes due to its heterogeneity, angiogenic activity, and invasiveness. Src family kinases (SFKs) play a crucial role in GBM progression, making them attractive targets for drug development. Here, we show results about the pharmacological profile of a new prodrug synthesized from a Src inhibitor, **SI306**. **Methods**: Three-dimensional-bioprinted GBM cell models were used in predicting the antitumor activity of the prodrug **SI306-PD2** with respect to its precursor, **SI306**. **Results**: Since the prodrug releases the active inhibitor through the cleavage by specific enzymes, **SI306-PD2** was analyzed for stability and release kinetics in various media, including fetal bovine serum (FBS), which is normally used in cell culture. In comparison to **SI306**, **SI306-PD2** demonstrated higher solubility in water, higher permeability across gastrointestinal and blood–brain barrier membranes, and the ability to release the drug in the presence of FBS progressively. In the 2D GBM cell model, using U87 and U251 cell lines, both compounds similarly reduced tumor cell viability. In 3D-bioprinted cell models, in the presence of an FBS-free medium, **SI306-PD2** exhibited a more effective antitumor activity compared to **SI306**, reducing the proliferation and diameter of U251 spheroids grown within the bioprinted scaffold in a statistically significant manner. The analysis of proteins extracted from 3D scaffolds confirmed that **SI306-PD2** inhibited Src activation more efficiently than **SI306**. **Conclusions**: Our study suggests that, when tissue permeability represents a discriminating characteristic, bioprinted cell models can provide a valid alternative for studying the cytotoxicity of new antitumor compounds. This approach has permitted us to ascertain the potential of the prodrug **SI306-PD2** as a therapeutic agent for GBM, demonstrating better tissue penetration and antiproliferative efficacy compared to the precursor compound **SI306**.

## 1. Introduction

The Src family kinases (SFKs) constitute a large cytosolic tyrosine kinase family orchestrating fundamental biological processes, such as growth, cell differentiation, metabolism, migration, and apoptosis. Docking interactions with many directing partners and the phosphorylation status of two important tyrosine residues closely govern the activity of the Src kinase, the prototypal member of SFK, at the cellular level [1]. The successful accomplishment of these regulatory mechanisms is necessary to avoid the oncogenic transformation of Src, leading to its constitutive activation and promoting neoplastic growth. An active Src kinase was found in several human tumors, attracting researchers to the development of specific targeted drugs. To date, tyrosine kinase inhibitors represent a well-known therapeutic tool, and they are used in the clinic to treat different kinds of solid tumors, such as sorafenib, approved for hepatocellular carcinoma, and sunitinib, approved for renal cell carcinoma [2,3,4]. Crucially, aggressive glioma tumors, such as glioblastoma multiforme (GBM), the primary cause of death from CNS malignancies, have also been found to exhibit elevated Src activity [5]. GBM suffers from poor treatability, due to peculiar biological characteristics, including the high phenotypic heterogeneity, the intense angiogenic activity, and the invasive ability towards the surrounding normal brain tissue. These characteristics suggest the existence of a tight capacity of interaction with the tumor microenvironment, rendering GBM potentially dependent on the Src signaling network that indeed plays a key role in regulating tumor microenvironment plasticity [6,7]. However, the clinical translation of the antitumoral activity of Src inhibition in solid tumors does not always confirm expectations [8]. Major hurdles during the development of kinase inhibitors are generally represented by the onset of drug resistance, low cell permeability, and poor pharmacokinetic properties [9]. To increase features such as tissue distribution and effectiveness, and reduce organ-specific toxicity, several strategies could be adopted in an early preclinical development phase, such as drug-delivery systems. In this context, the design of a prodrug represents a successful strategy to deliver the active compound and improve its biological activity.

**Figure 1:**
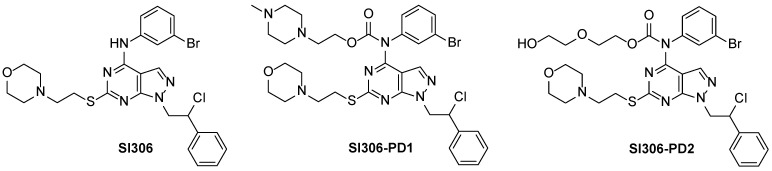
Structures of **SI306** and its prodrugs **SI306-PD1** and **SI306-PD2**.

In recent years, our research group was intensely involved in identifying new pyrazolo[3,4-*d*]pyrimidines active against GBM by inhibiting Src. Among them, the pyrazolo[3,4-*d*]pyrimidine **SI306** (K*_i_* = 0.13 µM) showed activity in GBM cell lines and strongly arrested U87MG xenograft growth in nude mice in combination with radiotherapy and exhibited a promising delivery to the brain [10]. However, **SI306**’s suboptimal water solubility has prompted the development of prodrugs **SI306-PD1** and **SI306-PD2**, with a potentially increased pharmacological profile (Figure 1). In fact, compared to parent pharmaceuticals, prodrugs demonstrate better hydrolysis in human and murine blood, as well as a greater capacity to cross cell membranes, according to ADME tests. Furthermore, in an orthotopic GBM model, **SI306-PD1** demonstrated greater efficacy than **SI306** [10]. With its poly(ethylene glycol) (PEG) chain, **SI306-PD2** (Appendix A) was synthesized to effectively modulate drug delivery and was active in U87 and CAS-1 GBM cells [11]. Its anti-survival effect was linked to a decrease in Src activation and the expression of EGFR-vIII and WT in U87MG and CAS-1 cells, respectively [12]. However, standard in vitro assays did not permit the appreciation of significant difference in **SI306-PD2** cytotoxic activity with respect to **SI306**. Indeed, the preclinical investigation of new drugs often fails to generate an accurate prediction of their clinical merit. Three-dimensional-bioprinted cell cultures promise a new tool in replicating cancer growth in vitro, and they can potentially improve how we develop new drugs, personalizing anticancer therapies in a clinically translatable manner. In particular, the use of hydrogels in 3D culture represents a feature able to improve our prediction of drug absorption. The three-dimensional printing of alginate/gelatin matrices is a versatile technique for creating complex transparent biomimetic structures with spatial precision [13]. In addition, the alginate/gelatin hydrogel forms a complex and stable matrix that allows for the formation of tumor spheroids, in a growth model resembling some characteristics of solid tumors in vivo, such as cell morphology, proliferation, oxygenation, and drug uptake. For these reasons, we decided to further investigate the **SI306-PD2** cytotoxic capacity, in comparison with **SI306**, using 3D cell models that could better recapitulate the in vivo situation and reveal the differences in ADME characteristics.

## 2. Materials and Methods

### 2.1. Chemicals and HPLC-UV/MS Method

Reagents and solvents for chemical synthesis were purchased from Sigma-Aldrich Srl (Milan, Italy) and Carlo Erba Reagents Srl (Milan, Italy). The Milli-Q ultrapure water purification system (Millipore, Milford, MA, USA) was used to obtain the distilled water for the experiment. The Agilent 1260 LC/MSD VL system (G1946C) (Agilent Technologies, Palo Alto, CA, USA) was used to perform the chromatographic analysis. It consisted of a 6130 series mass spectra detection (MSD) single-quadrupole instrument with the orthogonal spray API-ES (Agilent Technologies, Palo Alto, CA, USA), a vacuum sol-vent degassing unit, a binary high-pressure gradient pump, and a 1260 series UV detector. Nitrogen served as a drying and nebulizing gas. A Phenomenex Kinetex EVO C18—100 Å (150 mm × 4.6 mm, 5 μm particle size) was utilized for chromatographic separation at room temperature (RT), and gradient elution with a binary solution was carried out as shown in Table 1.

### 2.2. Cell Culture Medium, Fetal Bovine Serum, and Plasma Stability Assay

Compounds were solubilized in DMSO and were incubated at 37 °C under shaking in the presence of Dulbecco’s modified Eagle medium (DMEM) plus 10% fetal bovine serum (FBS), or absolute FBS, or human plasma (55.7 µg protein/mL). Stability tests in FBS and plasma were performed using HEPES buffer (25 mM, 140 mM NaCl, pH 7.4) as a mixing solvent. Samples were collected in a time-course way (from 0 to 24 h), treated with cold ACN to stop degradative reactions, and then spun down at 5000 rpm for 10 min. Supernatants were analyzed for the quantification of the compounds (**SI306-PD2**, **SI306** released from prodrug and **SI306**) using the HPLC-UV/MS method, as described above.

Data were plotted using GraphPad Prism 8.0 (GraphPad Software Inc., San Diego, CA, USA), and the half-life value (t_1/2_) was calculated with the following formula:

t 12=0.693b

where *b* is the slope found in the linear fit of the natural logarithm of the fraction remaining of the parent compound vs. incubation time.

### 2.3. Parallel Artificial Membrane Permeability Assay (PAMPA)

After being first dissolved in DMSO, the compounds were diluted 1:1 *v*/*v* using phosphate buffer (PBS 10 mM, pH 7.4). Because of their low and high apparent permeability across the phospholipidic bilayer, atenolol and propranolol were employed as reference molecules. The gastrointestinal (GI) and blood–brain barrier (BBB) phospholipidic bilayers were mimicked by covering each well of the filter plate with either a 10%_*w*/*v*_ CHCl_3_/dodecane brain polar lipid solution or a 1%_*w*/*v*_ L-α-phosphatidylcholine solution (PC) in dodecane. After placing a DMSO/PBS 1:1_*v*/*v*_ combination solution in the acceptor plate, the sandwich was gently shaken and incubated at room temperature for four hours. Samples were collected from the upper and bottom plates at the end time point, and the LC-UV/MS method was used for analysis. As previously reported, the apparent permeability (P_app_ cm/s × 10^−6^) was quantified [14].

### 2.4. Cell Culture

The human glioblastoma cell line U87 MG (U87) was originally isolated from a female patient with a malignant glioma (ECACC). The human glioblastoma cell line U251 MG (U251) was derived from a 75-year-old male patient with a malignant glioblastoma (ECACC). DMEM high-glucose growth medium (Euroclone, Milan, Italy), supplemented with 10% FBS (Euroclone), 200 mM L-glutamine (Euroclone), 100 IU/mL penicillin, and 100 µg/mL streptomycin (Euroclone) comprised the culture medium used for cell propagation at a controlled temperature and atmosphere, at 37 °C, 95% O_2_, and 5% CO_2_. Cell lines underwent regular testing for mycoplasma using the PCR method and identity verification was performed using DNA profiling.

### 2.5. 3D-Bioprinted Cell Model

A solution of 2% alginate and 8% gelatin (bio-ink) made up the hydrogel utilized for bioprinting. After 15 min of UV light exposure, the powders were dissolved in sterile DPBS in a magnetic stirrer set at 50 °C with laminar flow. After being made, bioink was put in sterile syringes and kept at 4 °C until it was needed. Bioink had been equilibrated at 37 °C prior to the bioprinting process. After that, 2.7 mL of bioink was added to 20 × 10^6^ cells that had been resuspended in 300 µL of growth media. Lastly, a disposable cartridge containing a cell/hydrogel suspension was equilibrated in a temperature-controlled printhead for 30 min at 29 °C. The BIO-X bioprinter (Cellink, Gothenburg, Sweden) was used to print in a 12-well plate at a speed of 5 mm/s and a dispensing pressure of 50 kPa, while the temperature was adjusted to 18 °C. Before adding culture media, three-dimensional constructs were crosslinked using CaCl_2_ for ten minutes and BaCl_2_ for three minutes. After 8 days, 3D structures, after evaluating cell viability, were treated for 72 h with **SI306** and **SI306-PD2** at selected concentrations and in different conditions: in complete DMEM high-glucose growth medium and DMEM-high glucose growth medium without FBS (*w*/*o*). Fiji ImageJ software (version 2.16.0/1.54p, https://imagej.net/software/fiji/downloads, accessed on 20 May 2025) was used to perform measurements of 3D models’ diameters on images acquired using phase contrast microscopy. A hemocytometer was used to set a scale for normalized measures and to convert real optic distance in pixels. At least five different optical fields were acquired, and the diameter was calculated for at least 100 different cell aggregates in each experimental point.

### 2.6. Cell Viability Assay

For the treatment of 2D cultures, U87 and U251 cell lines were initially seeded at a density of 2.5 × 10^4^ cells/mL in a 24 multiwell with a complete DMEM high-glucose growth medium. The viability test was started 24 h after cell plating and performed for 72 h. According to the experimental plan, the tests were performed in the presence of a complete DMEM high-glucose growth medium or, to avoid the serum-dependent degradation of prodrug, in an FBS-free DMEM high-glucose growth medium that was supplemented with 200 mM L-glutamine, 100 IU/mL penicillin, and 100 µg/mL streptomycin. Cell viability in 2D and 3D models was assessed using the cell-permeable resazurin-based solution PrestoBlue (Thermo Fisher, Waltham, MA, USA). After incubating the cells in a 1:10 ratio with the colorimetric reagent for two hours in the dark, the spectrophotometer readings at 570 and 600 nm were utilized as a relative indicator for cell viability.

### 2.7. Western Blot

Cells were subjected to total protein extraction using a ready-to-use cell lysis buffer (Euroclone, Milan, Italy), supplemented by a Protease/Phosphatase Inhibitor Cocktail (Euroclone), diluted 1:100. Cell lysis was further facilitated by three cycles of sonication in an ultrasonic bath (JSP, Los Angeles, CA, USA). After that, nuclei and large cellular debris were removed from the cell lysates by centrifuging them for 10 min at 300× *g*. Proteins were dosed using the Bradford Dye Reagent assay (Bio-Rad, Hercules, CA, USA). A 10% sodium-dodecyl sulphate polyacrylamide gel electrophoresis (SDS-PAGE) was performed on each sample, with Sharpmass VII protein molecular markers (Euroclone, Milan, Italy). Proteins were blotted to Amersham Protran 0.2 μm nitrocellulose membranes (Cytiva Europe, Freiburg, Germany) at 350 mA for 90 min. Then, membranes were blocked for one hour at room temperature using 10% non-fat dry milk (PanReac AppliChem, ITW Reagents, Milano, Italy) in Tris-buffered saline (Bio-Rad) at pH 7.4 with 20 mM Tris, 500 mM NaCl, and supplemented by 0.05% Tween 20 (Bio-Rad). Blotted proteins were probed for one hour at room temperature using the following antibodies, according to the manufacturer’s recommended dilution: anti-Src (36D10), anti-Phospho-Src (Thr416) (E6G4R), anti-GAPDH (0411), and HRP-conjugated anti-rabbit IgG or anti-mouse IgG as the secondary antibody (all from Cell Signaling Technology, Danvers, MA, USA). The protein bands were seen using the Chemidoc XRS system (Bio-Rad) to acquire the optical signals generated by the HRP-chemiluminescence reaction (Amersham, Buckinghamshire, UK), and Imagelab software (Bio-Rad, version 5.2.1 build 11) was used to digitally analyze them in order to calculate the band molecular weight and density.

### 2.8. Data Analysis and Statistics

Data were imported into Excel or GraphPad Prism software (version 6.01) and elaborated as the mean ± standard deviation (SD) of at least three independent experiments. The calculation of IC_50_ values with 95% confidence intervals was performed in GraphPad Prism software using a parameter nonlinear fit of the log-dose vs. response elaboration of data. The *p* value of the log-dose vs. response was calculated with a two-tailed Mann–Whitney test for unpaired data. The statistical significance between two series of measures, including stability and PAMPA (both GI and BBB) experiments, was calculated with the parametric Student t test, considering a significant *p* value lower than 0.05.

## 3. Results

### 3.1. Evaluation of SI306 and SI306-PD2 Stability and SI306 Release from Prodrug

**Figure 2:**
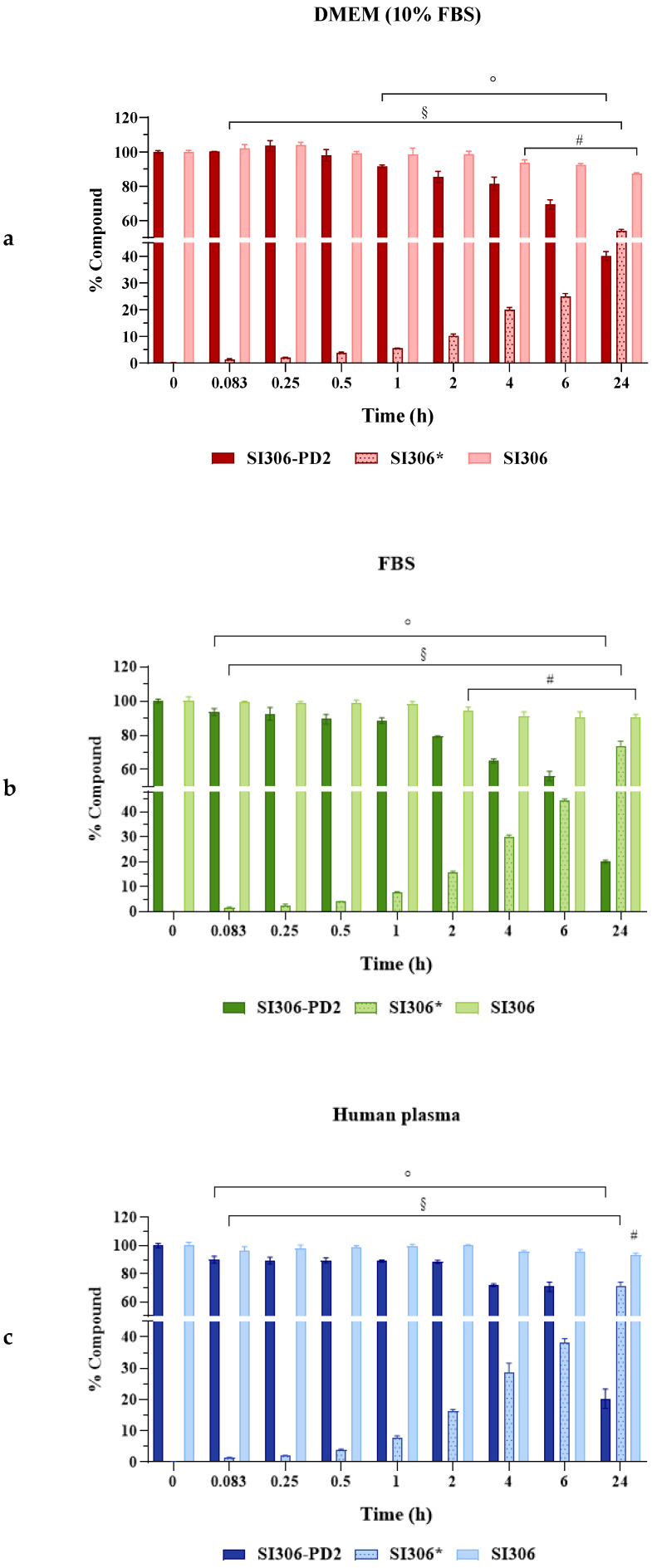
Stability/release studies of **SI306-PD2** (darker columns), SI306* (active compound released from **SI306-PD2**, lighter pointed columns) and **SI306** (lighter columns) in presence of (**a**) DMEM (10% FBS), (**b**) FBS, and (**c**) human plasma. The values represent the mean ± SD. The experiments were run in triplicate. ° *p* < 0.05 at each time point vs. time 0 of **SI306-PD2**. ^§^ *p* < 0.05 at each time point vs. time 0 of **SI306** released from **SI306-PD2**. ^#^ *p* < 0.05 at each time point vs. time 0 of **SI306**.

As a preliminary approach to evaluate their pharmacological potential, **SI306** and its prodrug **SI306-PD2** were tested and compared in terms of stability/release in human plasma. In addition, to correctly interpret the in vitro behavior of compounds, the stability/release was also evaluated in the presence of FBS used as an additive in culture medium (DMEM). Both compounds were incubated at a fixed concentration with DMEM (10% FBS), FBS, and human plasma at different time points (from time 0 to 24 h). **SI306** demonstrated a high stability in the presence of all different media, as reported in Table 2 and Figure 2. Indeed, after 24 h of incubation, the percentage of unmodified **SI306** was about 90% in all media (87.39%, 90.02%, and 93.12% in DMEM (10% FBS), FBS, and human plasma, respectively); moreover, these data were confirmed by the half-life (t_1/2_) values exceeding 100 h. The statistical analysis validated the t1/2 results, revealing a significant difference between SI306-PD2 and SI306 in all conditions (*p* < 0.05).

Regarding compound **SI306-PD2**, data clearly indicate its capability to release the active compound **SI306** over time. As a prodrug, **SI306-PD2** was confirmed to be more susceptible to the degradative action of FBS and plasmatic proteins, which hydrolyzed the carbamate and allowed for the release of **SI306**; indeed, when incubated in presence of DMEM added with 10% of FBS, the percentage of **SI306-PD2** remained stable for up to 1 h (91.48%), then significantly collapsed to around 70% after 6 h, and finally to 40.07% after 24 h of incubation (*p* < 0.05). As the prodrug was degraded, a greater amount of **SI306** was released in a significant way, starting from 0.083 h. The same trend has been appreciated when **SI306-PD2** was incubated in presence of FBS and human plasma; in both cases, due to the higher concentration of proteins used in the assays, the percentages of unmodified **SI306-PD2** resulted in a lower value than that obtained in DMEM with only 10% of FBS (Table 2, Figure 2). Also, in these cases, in the range of 0–1 h, a seven-fold increase in the concentration of **SI306** released from the prodrug was detected, accompanied by a significant reduction of **SI306-PD2** stability (around 90% after 1 h, *p* < 0.05); then, the percentage of the unmodified prodrug was about 20% after 24 h. According to percentages of stability obtained for **SI306-PD2** and **SI306**, the half-life values of the prodrug were shorter, but still significant, than those obtained for **SI306**; indeed, in the presence of DMEM (10% FBS) **SI306-PD2** was stable up to 18.09 h before 50% of the molecule degraded and released **SI306**, while a shorter time was required in the presence of FBS and human plasma (10.65 and 10.88 h, respectively).

### 3.2. Permeability Through Biological Membranes

To pre-evaluate pharmacokinetic properties, the ability to cross biological membranes was assessed using GI and BBB PAMPA assays. As reported in Table 3, **SI306** was confirmed to have a moderate apparent permeability (P_app_ 5.93 ± 0.38), as previously reported [15]. Moreover, its hydrophobic nature can also be confirmed by the high affinity towards lipidic membranes; indeed, this compound was characterized by a significant membrane retention (MR% 60.09 ± 3.38).

On the contrary, the prodrug **SI306-PD2**, endowed with the introduction of a PEG chain, demonstrated a significant increase in the apparent permeability across the membrane up to 18.45 ± 2.32, and simultaneously with a reduction in the percentage of membrane retention (MR% 5.26 ± 2.70) and in the predisposition to remain entrapped in the lipidic bilayers. When the phospholipid membrane was replaced by one mimicking the BBB, **SI306** and **SI306-PD2** slightly improved their apparent permeability to 8.44 and 19.51 × 10^−6^ cm/s, respectively, and consequently almost halved the percentages of membrane retention (MR% 39.95 and 3.60, respectively) with respect to the GI ones. The statistical analysis validated the P_app_ and MR% data, showing a significant difference (*p* < 0.05) between the GI and the BBB permeability results of **SI306-PD2** and **SI306**.

### 3.3. Antiproliferative Effect of SI306 and SI306-PD2 in 2D Cell Models

**Figure 3:**
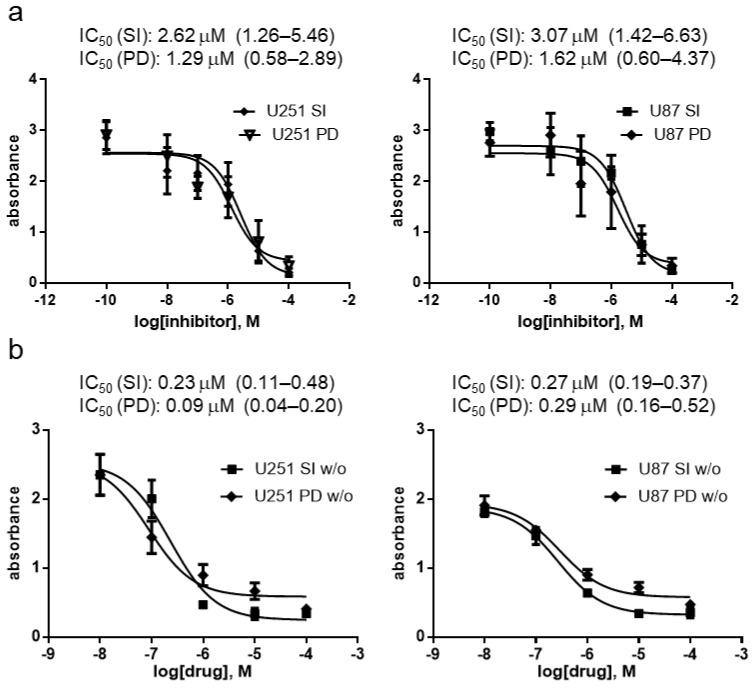
Viability assay of U251 and U87 glioblastoma cell lines treated for 72 h with increasing concentration of **SI306-PD2** (PD) and **SI306** (SI) in the presence of 10% FBS-DMEM (**a**) or FBS-free DMEM (*w*/*o*) (**b**). On top of each graph, the resulting IC_50_ (μM) with confidence interval is shown. The values are the mean of three different measures ± SD. *p* values: (**a**) (left) > 0.999; (right) = 0.788; (**b**) (left) = 0.587; (right) = 0.413.

Based on the previous data, the antiproliferation activity of **SI306** and **SI306-PD2** was evaluated on 2D GBM cell models. According to standard 2D cell culture, GBM cell lines (U87 and U251) were treated for 72 h with increasing concentrations (0.1-1-10-100 µM) of **SI306** and **SI306-PD2** in the presence of DMEM medium with or without 10% FBS (FBS-DMEM and FBS-free DMEM). Control cells were cultured with a percentage of the DMSO vehicle (<0.1%) corresponding to the highest concentration of the compounds. At the endpoint, viability was assessed using the PrestoBlue colorimetric assay, and the resulting absorbance subtracted from the blank was used as a relative surrogate for cell viability. Both compounds induced a dose-dependent reduction in viable U87 and U251 cells compared with control cells in both FBS-DMEM and FBS-free DMEM conditions (Figure 3). In detail, the IC_50_ curves in FBS-DMEM were very similar for all compounds and in the two cell lines (Figure 3a); the anti-survival effect was confirmed in the micromolar range against U87 cells and then assessed in the second GBM cell line, U251. Also, in this case, derivatives showed IC_50_ values between 1.29 and 2.62 µM. The treatment in the FBS-free medium demonstrated a lower IC_50_ value with respect to experiments in FBS-DMEM, with a reduction of about 10-fold (Figure 3a,b). Although no significant difference was appreciated when U251 and U87 cells were treated with **SI306** in the FBS-free medium (the IC_50_ values were comparable), a higher response to **SI306-PD2** with respect to **SI306** was detected in U251 cells, as indicated by the lower IC_50_ values (0.09 µM vs. 0.23 µM). However, the statistical analysis did not reveal a significant difference between compounds in any conditions (*p* > 0.05).

### 3.4. Antiproliferative Effect of SI306 and SI306-PD2 in 3D Cell Models

**Figure 4:**
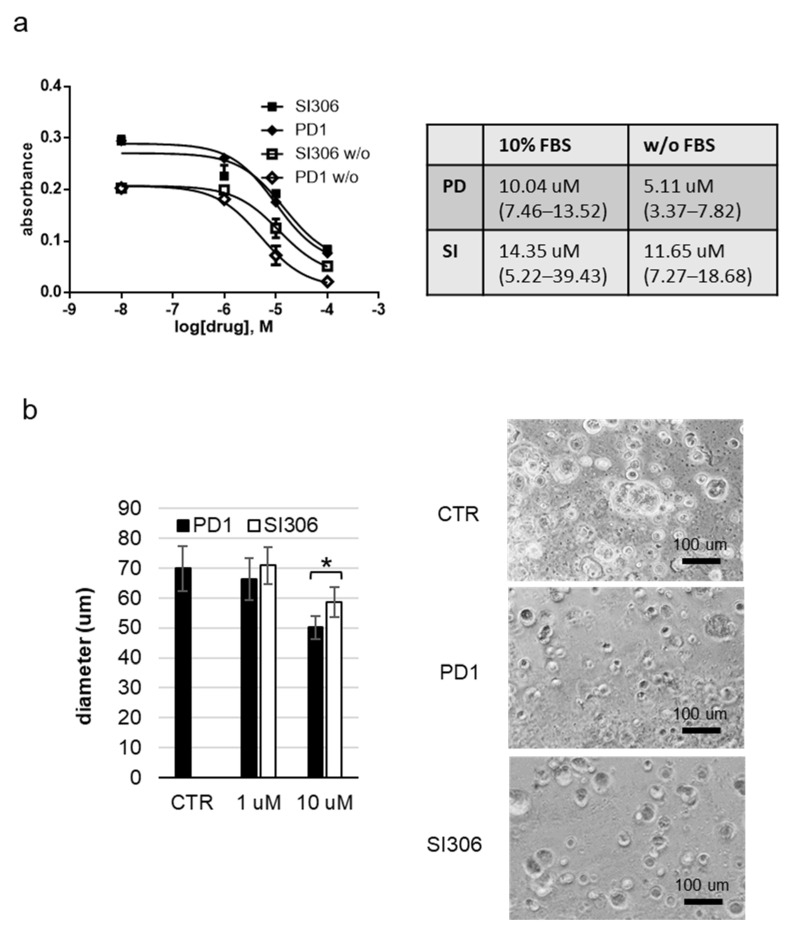
Evaluation of the antiproliferative effect of inhibitors in the bioprinted U251 cell model. (**a**) Viability assay of U251 glioblastoma cell line treated for 72 h with increasing concentration of **SI306-PD2** (PD) and **SI306** (SI) in the presence of 10% FBS-DMEM or FBS-free DMEM (*w*/*o*). The resulting IC50 values (μM) and the confidence intervals are reported in the table. The values are the mean of three different measures ± SD. *p* values: SI vs. PD = 0.971; SI *w*/*o* vs. PD *w*/*o* = 0.771. (**b**) Bioprinted cell cultures treated for 72 h with 1 and 10 μM of **SI306-PD2** (PD) and **SI306** (SI) in the presence of FBS-free DMEM were observed using microscopy and the diameter of cell aggregates was measured. The mean value from 100 measurements for each experimental point (±SD) is reported in the histogram and endpoint representative images from control culture and cultures treated with 10 μM of **SI306-PD2** (PD) and **SI306** (SI) are shown in the right panel. Statistical significance between experimental series is marked with an asterisk (*p* < 0.05).

The cytotoxic activity of **SI306** and **SI306-PD2** was also evaluated in 3D GBM cell models through a bioprinting procedure using alginate/gelatin hydrogel (details in M&M). The 3D-bioprinted culture demonstrated a greater resistance to the cytotoxic action of Src inhibitors with respect to bidimensional cultures, with IC_50_ values measured in tumor spheroids being up to about 50-fold higher than in cell monolayers. This evidence is in accordance with our previous results obtained with dasatinib, using similar experimental procedures, and suggests an increased difficulty in drug penetrance in 3D models. However, 3D cell models could offer useful information about the drug uptake in physiological conditions. Thus, the U251 cell line that demonstrated the highest sensitivity to inhibitors was utilized to perform a viability test in 3D culture conditions. U251 cells in the 3D structures, when observed using phase-contrast microscopy, were demonstrated to form spheroidal aggregates with an increasing diameter over time. Eight days after bioprinting, U251 3D-bioprinted structures were treated for 72 h with increasing concentrations of **SI306-PD2** and **SI306** in the presence of 10% FBS-DMEM or FBS-free DMEM. Control 3D-bioprinted structures were grown with a percentage of the DMSO vehicle (<0.1%_*v*/*v*_) corresponding to the highest concentration of the compounds. At the endpoint, viability was assessed using the PrestoBlue colorimetric assay. Both compounds induced a reduction in the number of viable U251 cells, with higher IC_50_ values with respect to the 2D cell model in both the 10% FBS-DMEM and FBS-free DMEM. The **SI306-PD2** treatment was able to reduce the U251 proliferation with a similar efficacy with respect to **SI306** in the presence of FBS, while the **SI306-PD2** IC_50_ value was about half with respect to the **SI306** IC_50_ value when cells were cultured without FBS (5.11 µM vs. 11.65 µM, Figure 4a).

The biological effect of treatment in the FBS-free DMEM was also evaluated by measuring the mean diameter of U251 spheroids in the hydrogel at the endpoint. After 72 h of treatment with 1 and 10 µM of **SI306-PD2** and **SI306**, the 3D cell cultures were observed under a phase-contrast microscope and the diameter of cell aggregates was measured. A significant difference in the mean diameter of the treated U251 spheroids with respect to the control was detected in the presence of 10 µM of the two compounds, and in this condition, the treatment with **SI306-PD2** resulted in a significant reduction in spheroid diameter with respect to **SI306-treated** cells (Figure 4b).

### 3.5. Evaluation of Src Activation in Bioprinted Cells

**Figure 5:**
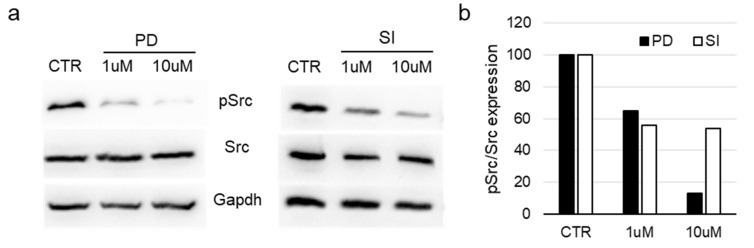
Evaluation of Src activation in bioprinted cell model. (**a**) Total cell lysates from U251 cells grown in an alginate/gelatin bioprinted scaffold were subjected to Western blot analysis. Total cell lysates were examined for the expression of pSrc (Tyr416), total Src, and GAPDH (loading control) after bioprints were treated for 24 h with 1 or 10 μM **SI306-PD2** (PD) or **SI306** (SI) in culture media devoid of serum. (**b**) Protein bands’ relative expression levels were examined in blots, and the histogram displays the pSrc/Src ratio normalized for control values.

To further validate our hypothesis that **SI306-PD2** may better reach the biological target and induce a superior antitumoral effect than **SI306** in 3D cell cultures, Src activation was analyzed using Western blot analysis in U251 cell lysates from bioprinted structures. Eight days after bioprinting, the structures were treated for 24 h with 1 or 10 µM **SI306-PD2** or **SI306** in FBS-free DMEM. Control structures were maintained with a percentage of the DMSO vehicle (<0.1%_*v*/*v*_), corresponding to the highest concentration of compounds. At the endpoint, total cell lysates were analyzed for the expression of Src, of its active form pSrc (Tyr416), and GAPDH as the loading control. Both compounds induced an evident reduction in the active form of Src, starting from 1 µM compared with the experimental control condition (Figure 5a). The ability to inhibit Src activity by **SI306-PD2** and **SI306** was evaluated using a densitometric analysis of the Western blot bands, considering the pSrc/Src ratio normalized to control values (Figure 5b). This analysis suggested a higher ability of the prodrug to inhibit the generation of phosphorylated Src than the **SI306** compound at a concentration of 10 µM.

## 4. Discussion

Solid cancers have complex and heterogeneous structures that play an important functional role in their therapeutic response; thus, the development of in vitro predictive cell models represents a key challenge in drug discovery. In our 3D models, we used a biological source-derived hydrogel containing alginate and gelatin that combines the favorable mechanical properties of alginate to facilitate the bioprinting process and the stability of the molecular network, with the physiological supportive role of collagen. Additionally, alginate hydrogels are composed of crosslinked polymers that create swelling matrices that mimic the biological characteristics of extracellular matrices and can absorb a lot of water. The utilization of bioprinted cell models in our experimental design aimed to clarify the presumable difference in the antitumoral activity of the prodrug **SI306-PD2** compared to the parental Src-inhibitor **SI306**, since bi-dimensional cultures failed to demonstrate any significant variation in their antiproliferative capacity.

Interestingly, comparing the cytotoxic activity of compounds in the U251 3D culture, we appreciated a higher antitumoral effect of **SI306-PD2** vs. **SI306** only in the FBS-free medium, with a lower IC_50_ of **SI306-PD2** with respect to **SI306** (5.11 vs. 11.65 μM). The analysis of other biological parameters confirmed the superior toxicity of **SI306-PD2** with respect to **SI306**, mainly when compounds were used at concentrations equal to or higher than IC_50_ values. Indeed, the diameter of the U251 spheroid at the endpoint of 72 h following the drug treatment (10 µM) in the FBS-free medium was smaller for **SI306-PD2** than for **SI306**. The interpretation of these results could be conducted according to data about the stability/release and permeability of the compounds. As expected, stability studies demonstrate that, in the presence of FBS, **SI306-PD2** progressively delivers the biologically active compound **SI306**. As appreciated from the data reported in Table 2, **SI306** was constantly released from the prodrug starting from time 0, reaching the 50% of the release point in 24 h. In parallel, we confirmed that **SI306**, during the same time interval, resisted the possible degradative action of the proteins contained in the FBS or human plasma. Taken together, these results suggest that the foreseen advantage in solubility of **SI306-PD2** with respect to **SI306** is halved during the first 24 h of culture in the presence of FBS. The importance of the stability of **SI306-PD2** is further sustained by results obtained in permeability tests. According to these data, we may assume that the delivery of **SI306** throughout the prodrug was an efficient strategy, since **SI306-PD2** demonstrably reached the intracellular target by crossing cellular membranes through passive diffusion mechanisms better than **SI306**. Importantly, according to our analysis, **SI306-PD2** was predicted to also more easily cross the blood–brain barrier with respect to **SI306**, reinforcing its therapeutic value in the field of CNS tumors. The capacity to cross the cell stratum is particularly important when a drug is tested in a 3D model. The spheroid growth of cells contributes to conferring drug resistance through a limitation in the penetration into the core of the cell aggregate. We hypothesize that the absence of FBS in the medium contributes to slowing the release of **SI306** from the prodrug and allows for an easy and progressive diffusion of the **SI306-PD2** through the cell spheroid, resulting in a favorable IC_50_ value for the prodrug **SI306-PD2**. These data followed the trend barely appreciated in 2D models, suggesting that the experimental condition that prevented the activation of **SI306-PD2** outside the cell (serum-free culture medium), combined with the greater ability to cross biological membranes without becoming trapped in them, could be observed only in 3D cell models.

Although the spheroid culture represents a useful tool in understanding the drug uptake and diffusion within tissues, the analysis of the distribution of a drug metabolite in cell spheroids, in the absence of a tracking dye, is challenging. Mass spectrometry revealed that the prodrug irinotecan was found in the center of tumor spheroids, indicating a greater capacity to penetrate tumor tissues [16,17]. In our experimental approach, we were able to test the activity of the compounds by evaluating the status of the activation of the targeted Src, collecting protein lysates from all cells in the spheroids 24 h after the treatment. This approach permitted having a robust surrogate of the capacity of penetration of the compounds, comparing spheroids in a situation that was not yet influenced by a cytostatic or cytotoxic effect, and evaluating the functional target responsible for the biological effects. Since enzymatic studies previously demonstrated that several prodrugs of pyrazolo[3,4-*d*]pyrimidines have a limited intrinsic inhibitory activity [10,15] and based on the improved apparent permeability of **SI306-PD2** compared to the active molecule **SI306**, we suggest that activation of the prodrug was accomplished within cells at different depths in the tri-dimensional cell aggregate.

Data collected in this study, thanks to the utilization of a 3D model approach, permit us to confirm the promising antitumoral characteristics of our prodrug, highlighting superior tissue penetration in parallel with the preservation of the same inhibitory activity compared to the parental drug. Further studies should permit us to understand the optimal in vivo administration to preserve the useful pharmacological characteristics of the prodrug until reaching the tumor mass.

## Figures and Tables

**Table 1:** Chromatographic parameters adopted for the LC-UV/MS method. ^a^ The detection was conducted in positive [M]^+^ mode of ionization.

Time (min)	% Eluent A H_2_O (FA 0.1%_*v*/*v*_)	% Eluent B ACN (FA 0.1%_*v*/*v*_)	Flow (µL/min)	Injection Volume (µL)	Wavelength (nm)	Scan Range (*m*/*z*) ^a^
0–1	95	5	600	10	254	100–2000
15–19	5	95				
20	95	5				

**Table 2:** Stability/release studies of **SI306-PD2** and **SI306** in the presence of DMEM (10% FBS), FBS, and human plasma. ^a^ Half-life (h) expressed as the amount of time it takes before half of the drug is hydrolyzed/degraded. ^b^ Percentages of **SI306** released from **SI306-PD2**. ° *p* < 0.05 at each time point vs. time 0 of **SI306-PD2**. ^§^ *p* < 0.05 at each time point vs. time 0 of **SI306** released from **SI306-PD2**. ^#^ *p* < 0.05 at each time point vs. time 0 of **SI306**. * *p* < 0.05 t_1/2_ of **SI306-PD2** vs. t_1/2_ **SI306** in the same biological matrix. Half-lives for **SI306** released from the prodrug were not calculated.

Time (h)	Stability/Release (% ± SD)					
	DMEM (10% FBS)		FBS		Plasma	
	SI306-PD2 (SI306) ^b^	SI306	SI306-PD2 (SI306) ^b^	SI306	SI306-PD2 (SI306) ^b^	SI306
0	100.02 ± 0.80 (0.11 ± 0.02)	100.11 ± 0.91	100.06 ± 1.13 (0.15 ± 0.03)	100.24 ± 2.48	101.03 ± 1.58 (0.13 ± 0.04)	100.50 ± 2.32
0.083	100.22 ± 0.10 (1.37 ± 0.23) ^§^	101.97 ± 2.32	93.64 ± 2.06 ° (1.55 ± 0.29) ^§^	99.67 ± 0.21	90.12 ± 2.45 ° (1.37 ± 0.23) ^§^	96.64 ± 2.74
0.25	103.59 ± 2.97 (2.06 ± 0.09) ^§^	103.96 ± 1.68	92.56 ± 3.74 ° (2.24 ± 0.73) ^§^	98.42 ± 1.19	89.41 ± 2.37 ° (2.06 ± 0.09) ^§^	97.99 ± 2.48
0.5	98.01 ± 3.40 (3.79 ± 0.31) ^§^	99.17 ± 1.10	89.39 ± 2.87 ° (4.00 ± 0.14) ^§^	98.49 ± 2.18	89.57 ± 1.82 ° (3.88 ± 0.34) ^§^	98.95 ± 1.18
1	91.48 ± 0.86 ° (5.55 ± 0.09) ^§^	98.52 ± 3.69	88.58 ± 1.66 ° (7.94 ± 0.14) ^§^	97.91 ± 1.79	89.05 ± 0.75 ° (7.67 ± 0.76) ^§^	99.48 ± 1.70
2	85.45 ± 3.20 ° (10.21 ± 0.63) ^§^	98.64 ± 1.79	79.26 ± 0.34 ° (15.82 ± 0.36) ^§^	93.95 ± 2.42 ^#^	88.55 ± 1.18 ° (16.29 ± 0.54) ^§^	99.92 ± 0.61
4	81.41 ± 3.94 ° (19.99 ± 0.81) ^§^	93.56 ± 1.79 ^#^	64.90 ± 1.21 ° (30.02 ± 0.66) ^§^	90.92 ± 2.68 ^#^	71.86 ± 1.03 ° (28.80 ± 2.84) ^§^	95.72 ± 0.76
6	69.38 ± 2.73 ° (24.95 ± 1.09) ^§^	92.30 ± 0.84 ^#^	56.13 ± 2.72 ° (44.32 ± 0.78) ^§^	90.51 ± 3.24 ^#^	70.80 ± 3.25 ° (38.02 ± 1.46) ^§^	95.54 ± 1.74
24	40.07 ± 1.70 ° (53.92 ± 0.90) ^§^	87.39 ± 0.41 ^#^	20.06 ± 0.66 ° (73.80 ± 2.65) ^§^	90.02 ± 2.28 ^#^	20.29 ± 3.07 ° (71.59 ± 2.45) ^§^	93.12 ± 1.70 ^#^
t_1/2_ ^a^	18.09 *	>100	10.65 *	>100	10.88 *	>100

**Table 3:** In vitro passive permeability of compounds **SI306** and **SI306-PD2** across GI and BBB membranes. ^a^ Apparent permeability (P_app_) reported in cm/s × 10^−6^; ^b^ Membrane retention %.

Cpd	PAMPA Assay			
	GI		BBB	
	P_app_ cm/s × 10^−6 a^	MR (% ± SD) ^b^	P_app_ cm/s × 10^−6 a^	MR (% ± SD) ^b^
SI306	5.93 ± 0.38	60.09 ± 3.38	8.44 ± 1.03 ^§^	39.95 ± 0.97 ^§^
SI306-PD2	18.45 ± 2.32 *	5.26 ± 2.70 *	19.51 ± 0.46 *^§^	3.60 ± 1.15 *
Atenolol	0.73 ± 0.06	53.58 ± 1.96	1.01 ± 0.20	21.03 ± 1.14
Propranolol	19.16 ± 0.37	8.89 ± 1.12	17.16 ± 0.31	10.73 ± 1.33

* *p* < 0.05 of P_app_ and MR of **SI306-PD2** vs. P_app_ and MR of **SI306** in the same GI or BBB PAMPA assay. ^§^ *p* < 0.05 of P_app_ and MR of **SI306-PD2** and **SI306** in GI PAMPA assay vs. those in BBB PAMPA assay.

**Figure A1:**
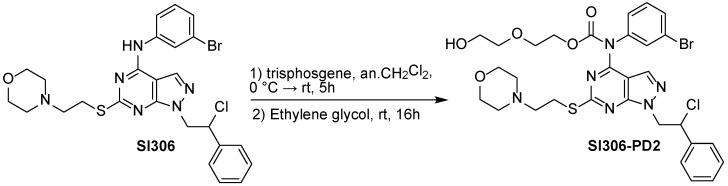
Synthesis of SI306-PD2.

## Data Availability

Most data generated or analyzed during this study are included in this article. The datasets and materials used and/or analyzed during the current study are available from the corresponding author on reasonable request.

## Appendix A

### Appendix A.1. Chemistry

We previously reported the synthesis of **SI306** and **SI306-PD1** [10]. Starting with **SI306**, **SI306-PD2** was created using a one-pot, two-step process. A pre-cooled solution of SI306 was first mixed with triphosgene, followed by ethylene glycol, and the reaction was agitated at room temperature to produce **SI306-PD2** with a good yield (Figure A1).

Every chemical that was sold commercially was acquired from Sigma-Aldrich in St. Louis, MO, USA. Molecular sieves (3 Å, 10% *m*/*v*) were used to dry DCM. Merck TLC silica gel 60 F254 was used for TLC. For the flash procedure, chromatographic purifications were carried out on columns filled with Merck silica gel 60, 23–400 mesh. A JEOL JNM ECZ-400S/L1 FT (400 MHz) was used to record the 1H-NMR and 13C-NMR spectra. At 0.00 ppm, chemical changes were observed in relation to tetramethylsilane. Thermo Scientific Flash 2000 was used to determine the elemental analyses for C, H, N, and S. The results were within ± 0.4% of the theoretical value. Every target compound had a purity of at least 95%, which was confirmed using an elemental analysis.

### Appendix A.2. Synthesis of 2-(2-Hydroxyethoxy)ethyl (3-Bromophenyl)(1-(2-chloro-2-phenylethyl)-6-((2-morpholinoethyl)thio)-1H-pyrazolo[3,4-d]pyrimidin-4-yl)carbamate (SI306-PD2)

A solution of triphosgene (0.45 mmol, 133 mg) in anhydrous CH_2_Cl_2_ (8 mL) was added dropwise to a mixture of NaHCO_3_ (2.25 mmol, 189 mg) and N-(3-bromophenyl)-1-(2-chloro-2-phenylethyl)-6-((2-morpholinoethyl)thio)-1H-pyrazolo[3,4-*d*]pyrimidin-4-amine **SI306** (0.45 mmol, 258 mg), in anhydrous CH_2_Cl_2_ (8 mL) precooled at 0 °C in an ice bath. After 30 min, the reaction was allowed to warm to room temperature and stirred for 5 h. Then, a solution of ethylene glycol (20.00 mmol, 1.9 mL) in anhydrous CH_2_Cl_2_ (8 mL) was added, and the mixture was stirred at room temperature for 16 h. Cold water (25 mL) was added, and the two phases were separated. The aqueous phase was extracted twice with CH_2_Cl_2_ (2 × 25 mL). The organic phase was dried over anhydrous Na_2_SO_4_ and evaporated. The crude oil was purified through two flash chromatographies on silica gel, eluting first with THF/EP 1:1 and then with ethyl acetate/acetone (1:1), affording the desired **SI306-PD2** as a pure light amber oil. Yield 54%. ^1^H-NMR (CDCl_3_): δ 2.48–2.57 (m, 6H, 2CH_2_N morph + NCH_2_CH_2_), 298–3.13 (m, 2H, CH_2_S), 3.47 (t, *J* = 8 Hz, 2H, HOCH_2_CH_2_O), 3.64–3.72 (m, 8H, 2CH_2_O morph + HOCH_2_ + COO-CH_2_CH_2_), 4.41 (t, J = 4 Hz, 2H, COO-CH_2_), 4.68–4.72 and 492–4.98 (2m, 2H, CH_2_N pyraz), 5.48–5.52 (m, 1H, CHCl), 7.16–7.18 (m, 1H Ar), 7.25–7.34 (m, 4H Ar), 7.41–7.43 (m, 3H Ar), 7.49–7.51 (m, 1H Ar), and 7.98 (s, 1H, H-3). ^13^C-NMR (CDCl_3_): δ NMR (101 MHz,) δ = 31.87, 53.45, 53.93, 54.32, 57.66, 60.29, 61.70, 66.58, 66.82, 68.74, 72.62, 76.85, 77.16, 77.48, 103.66, 122.36, 127.44, 127.56, 128.88, 129.17, 130.49, 131.33, 131.93, 135.39, 137.88, 140.98, 153.45, 154.27, 156.07, and 168.36. Anal. calcd. for C_30_H_34_N_6_O_5_SClBr: C 51.03, H 4.85, N 11.90, and S 4.54; found: C 50.77, H 5.19, N 11.57, and S 4.09.
